# Downregulation of RhoB Inhibits Cervical Cancer Progression and Enhances Cisplatin Sensitivity

**DOI:** 10.3390/genes15091186

**Published:** 2024-09-10

**Authors:** Weijiao Wang, Yubin Jia, Yuhuan Liu, Xiaofeng Lv, Lili Guo, Silu Meng, Changyu Wang

**Affiliations:** 1 Department of Obstetrics and Gynecology, Tongji Hospital, Tongji Medical College, Huazhong University of Science and Technology, Wuhan 430030, China; wangweijiao1004@163.com (W.W.); jiayubin0124@163.com (Y.J.); 15310460047@163.com (Y.L.); xiaofenglv@hust.edu.cn (X.L.); sunnylili11@126.com (L.G.); 2 Cancer Biology Research Center, Tongji Hospital, Tongji Medical College, Huazhong University of Science and Technology, 1095 Jiefang Ave, Wuhan 430030, China; 3 Department of Gynecologic Oncology, Women’s Hospital, School of Medicine, Zhejiang University, Hangzhou 310000, China

**Keywords:** cervical cancer, RhoB, prognosis, cisplatin

## Abstract

RhoB, a member of the Rho GTPase family, has been implicated in the malignant progression of various cancer types. However, its role in cervical cancer (CC) remains unclear. Therefore, this study aims to elucidate the biological function of RhoB in CC and its relationship with cisplatin sensitivity. We analyzed data from the TCGA, GTEx, and GEO databases, revealing that *RhoB* mRNA expression is downregulated in CC tissues compared to normal cervical tissues. The further analysis of the TCGA database and Tongji samples showed that CC patients with a high RhoB expression had a shorter overall survival (OS). Subsequently, we found that the knockdown of RhoB inhibited the proliferation, migration, and invasion of cancer cells, while increasing apoptosis. Through Western blot (WB) analysis, we found that knocking down RhoB resulted in an increased expression of the epithelial marker E-cadherin, while the levels of N-cadherin, MMP2, MMP9, Vimentin, and Snail1 were reduced. Additionally, *RhoB* mRNA expression was upregulated in CC tissues after chemotherapy compared to CC tissues before chemotherapy. In CC cells, RhoB expression increased with cisplatin concentration, and the IC50 value decreased following RhoB knockdown. Moreover, the knockdown of RhoB could enhance the cellular apoptosis triggered by cisplatin. This study demonstrated that RhoB plays an oncogenic role in CC and that its knockdown could enhance the sensitivity of CC cells to cisplatin.

## 1. Introduction

Cervical cancer (CC) is the fourth most prevalent malignant neoplasm among women worldwide [1]. A total of 604,127 new cases of CC and 341,831 related deaths were reported globally in 2020 [2]. A study indicated that the 5-year survival rates for CC were 90.9% for stage I, 71.0% for stage II, 41.7% for stage III, and only 7.8% for stage IV [3]. Patients with stage IB2-IIB CC can now benefit from a new treatment option that combines platinum-based neoadjuvant chemotherapy (NACT) with radical hysterectomy. This approach complements concurrent cisplatin-based chemotherapy, radiotherapy (CCRT), and brachytherapy [4]. Cisplatin is widely used as a chemotherapy agent for CC. However, due to increased drug efflux, genetic and epigenetic changes, and decreased drug accumulation, the resistance rate to chemotherapy drugs increases [5]. Despite advancements in treatment, the mechanisms driving resistance remain elusive, highlighting the urgent need to identify new therapeutic targets and strategies to improve cisplatin sensitivity in CC.

RhoB, a constituent of the Rho GTPase family, plays an important role in diverse cellular processes, including actin dynamics, cell migration, membrane trafficking, cell proliferation, DNA repair, and apoptosis [6,7,8]. Notably, RhoB has been implicated in fostering malignant phenotypes across various cancer types. For instance, in glioblastoma, the downregulation of RhoB expression prompted cell cycle arrest and apoptosis while reducing basal STAT3 activity [9]. Similarly, in skin tumors, RhoB emerged as a potent driver of tumor progression [10]. Additionally, the knockdown of RhoB was found to enhance radiotherapy sensitivity in colorectal cancer [11]. Despite these insights, the role of RhoB in CC is still elusive. Thus, this study aims to unravel the biological function of RhoB in CC and its association with cisplatin sensitivity.

## 2. Methods and Materials

### 2.1. Cell Lines and Culture

From the American Type Culture Collection (ATCC), we obtained the CC cell lines HeLa, SiHa, CaSki, and C-33A, as well as the normal cervix epithelial cells Hcer-Epic. We cultured them in Dulbecco’s Modified Eagle’s Medium (DMEM) at 37 °C with 5% CO_2_. Moreover, 10% fetal bovine serum (FBS), streptomycin (100 μg/mL), and penicillin (100 U/mL) were added to the DMEM medium.

### 2.2. Clinical Specimens

From Tongji Hospital, we recruited 89 CC samples. This study was approved by the Ethics Committee of Tongji Hospital, Tongji Medical College, Huazhong University of Science and Technology. All participants provided written informed consent before participating in this study.

### 2.3. RhoB Expression Analysis

To explore the *RhoB* mRNA expression level between normal tissues and CC tissues, the TCGA and GETx datasets were utilized. The further investigation of *RhoB* mRNA expression in CC tissues and normal cervix tissues was facilitated by downloading gene expression profiles from the Gene Expression Omnibus (GEO) database for GSE7803 [12], GSE9750 [13], GSE39001 [14], GSE52903 [15], GSE63514 [16], GSE63678 [17], and GSE89657 [18]. The number of normal cervix tissues and CC tissues of the TCGA and GTEx databases and 7 GEO datasets is listed in Supplementary Table S1.

### 2.4. Survival Analysis

The RNA-seq data and clinical information of squamous CCs (*n* = 252) were retrieved from the TCGA database. OS information was obtained from Tongji Hospital and the TCGA Pan-Cancer Clinical Data Resource [19]. The clinical characteristics of the cohorts used for survival analysis are detailed in Supplementary Table S2 [20]. For 252 squamous CCs from the TCGA database, the survival analysis was based on the median value of *RhoB* mRNA expression. For Tongji samples, the survival analysis was based on the histologic score (HScore) of immunohistochemistry (IHC). Survival curves were plotted by the Kaplan–Meier method, with statistical significance assessed by the Log-rank test. In the multivariate Cox analysis, factors were identified as independent risk factors for predicting CC if their *p*-value < 0.05. The analysis was performed with the survival and survminer R packages (version 4.3.1) and visualized using the forestplot R package (version 4.3.1).

### 2.5. Differentially Expressed Gene (DEG) Analysis

The Limma package was used to explore the DEGs between pre-NACT and post-NACT tissues, which were derived from Renji Hospital (SRP173984) [21,22]. Genes meeting the criteria of |LogFC| > 1 and adjust *p*-value < 0.05 were considered as DEGs.

### 2.6. Gene Ontology (GO) and Kyoto Encyclopedia of Genes and Genomes (KEGG) Analysis

Using the Spearman rank correlation test, we identified the top 500 genes that showed a positive correlation with *RhoB* expression. Next, these genes were analyzed for GO and KEGG analysis using the clusterProfiler R package [23]. The results were filtered based on the criteria of adjusted *p*-value < 0.05 and *q*-value < 0.05.

### 2.7. RT-qPCR

Total RNA was isolated from cultured cells using an RNA extraction kit (Vazyme, Nanjing, China) according to the instructions. To remove the genomic DNA, the isolated RNA was combined with RNase-free ddH_2_O and 4 × gDNA wiper Mix (Vazyme, Nanjing, China) at 42 °C for 2 min. After that, 5 × HiScript III qRT SuperMix (Vazyme, Nanjing, China) was added, and the mixture was subjected to reverse transcription into cDNA at 37 °C for 15 min, followed by 5 s of heating to 85 °C. When amplifying cDNA, we used Vazyme’s ChamQ Universal SYBR qPCR Master Mix. The mRNA expression levels were calculated using the threshold cycle (Ct) values, and the analysis was carried out using the Bio-Rad CFX Manager 3.1 software. The primers, both forward and reverse, that were utilized are listed in Supplementary Table S3.

### 2.8. IHC and Scoring

For the Detailed IHC procedure, we referred to a previously published article [24]. Two pathologists independently assessed the IHC scores without prior knowledge of the case details. The staining intensity was classified using a scale ranging from 0 to 3: There are different levels of staining, ranging from no staining to weak, moderate, and strong staining. The distribution of cells in each category was estimated from 0% to 100%. The HScore, a score ranging from 0 to 300, was calculated by multiplying the stain intensity by the percentage of cells stained at each level [25].

### 2.9. RNA Interference

RioBio (Guangzhou, China) synthesized siRNAs. Supplementary Table S3 lists the target sequences of siRNAs. Upon reaching a confluency of 40% to 50%, HeLa and CaSki cells were transfected at a concentration of 50 nM in six-well plates. At the outset, 250 µL of Opti-MEM was used to dilute 5 µL of Lipofectamine 3000 (Invitrogen, Los Angeles, CA, USA), and at the same time, 50 nM of siRNA was diluted in 250 µL of Opti-MEM. After mixing the two solutions, the transfection complex was left to rest at room temperature for 15 min before being applied to the cells. The culture media in the plates were replaced after 24 h. Efficient siRNAs were used in the subsequent functional studies.

### 2.10. Cell Proliferation and Colony Formation Assay

After 48 h of siRNA transfection, HeLa and CaSki cells were collected and plated in 96-well plates at densities of 3 × 10^3^/100 µL and 4 × 10^3^/100 µL, respectively. The cells were cultivated for 5 h, 24 h, 48 h, and 72 h. Afterwards, 10 µL of CCK-8 was added to each well and left to incubate in the dark at 37 °C for one to four hours. In order to measure the absorbance at 450 nm, the Spectra Max190 (Molecular Devices, Sunnyvale, CA, USA) was utilized. Each group was designed with three replicate wells, and the experiment was independently repeated three times.

In six-well plates, 1000 HeLa cells per well and 1500 CaSki cells per well were plated after 48 h of siRNA transfection. The cells were then cultured at 37 °C for 7–14 days. Colonies were scanned using an EPSON digital scanner (Suwa, Japan) after being treated with 4% paraformaldehyde and stained with crystal violet.

### 2.11. Flow Cytometry

After 48 h of siRNA transfection, CC cells were collected. Then, we stained cells using the AnnexinV-FITC apoptosis detection kit (BD, Franklin Lakes, NJ, USA) and subsequently analyzed using a Beckman flow cytometry.

### 2.12. Transwell Assay

We resuspended HeLa and CaSki cells in serum-free DMEM at varying densities (HeLa: 3 × 10^4^/200 µL for migration/invasion; CaSki: 6 × 10^4^/200 µL for migration/invasion). Then, we added the cell suspension (typically 200 µL) to the upper chamber of the 8 µm transwell (Greiner Bio-One, Frastanz, Austria) and added a medium containing a chemoattractant (20% FBS) to the lower chamber (typically 800 µL). For the invasion analysis only, we coated the upper chamber with 50 µL of Matrigel at a dilution of 1:19 (BD Biosciences, Franklin Lakes, NJ, USA). It was then incubated for 24 to 72 h. The cells in the upper chamber were gently wiped with a cotton swab and then washed twice with PBS after incubation. After that, the cells were fixed with 4% paraformaldehyde for 15 min. Following fixative removal, the cells were subjected to two rounds of PBS washing, stained with crystal violet for 30 min, followed by three rounds of PBS washing, swabbed to remove dye, and allowed to air-dry. Migrated or invaded cells were then imaged from three randomly selected microscopic fields per well. We repeated the experiment independently three times.

### 2.13. Cell Wound Healing

After 48 h of siRNA transfection, HeLa and CaSki cells were seeded in 6-well plates and allowed to grow until they reached 90%–100% confluence. The 200 µL pipette tip was used to make a scratch, and the cells were grown in a medium that did not contain serum. Microscopical observations were made at 0, 24, 48, 72, and 96 h after scratching. We used the lines on the back of the well as markers to photograph the scratch in multiple fixed areas for each well. We used ImageJ to measure the scratch area at different time points.

### 2.14. Western Blot (WB)

Equal amounts of proteins, typically in quantities of 20–40 µg, were subjected to SDS-PAGE electrophoresis and subsequently transferred to a PVDF membrane with a pore size of 0.45 µm. After that, 5% non-fat dry milk was prepared in Tris-buffered saline with 0.1% Tween-20 (TBST) and blocked for 1 h on a shaker at room temperature. Following this, we incubated the membranes overnight at 4 °C with gentle shaking using primary antibodies. The antibodies used are listed below: anti-RhoB (14326-1-AP, Proteintech, Wuhan, China), anti-β-actin (AC026, ABclonal, Wuhan, China), anti-E-cadherin (YT1454, Immunoway, Beijing, China), anti-N-cadherin (YT2988, Immunoway, Beijing, China), anti-Vimentin (YT4880, Immunoway, Beijing, China), anti-MMP2 (A19080, ABclonal, Wuhan, China), anti-MMP9 (A0289, ABclonal, Wuhan, China), and Snail1 (A11794, ABclonal, Wuhan, China). The antibodies were diluted with antibody dilution buffer (AntGene Corporation, Shanghai, China) at a ratio of 1:1000. Next, we incubated the membranes with secondary antibodies (Antgene Corporation, Shanghai, China) on a shaker at room temperature for 1 h. WesternBrightTM ECL (Advansta, Menlo Park, CA, USA) and ChemiDocTM Imaging System (Bio-Rad, Hercules, CA, USA) were used to capture the signals.

### 2.15. Statistical Analysis

R software (version 4.3.1) and GraphPad Prism (version 9.0) were utilized for statistical analysis. Differences between two groups were assessed with either a two-tailed Student’s *t*-test or a Wilcoxon test. The chi-squared test was used to evaluate the association between RhoB expression and clinical features, while correlations were calculated using the Spearman rank test. A *p*-value of less than 0.05 was statistically significant.

## 3. Results

### 3.1. The Expression of RhoB Is Downregulated in CC

**Figure 1:**
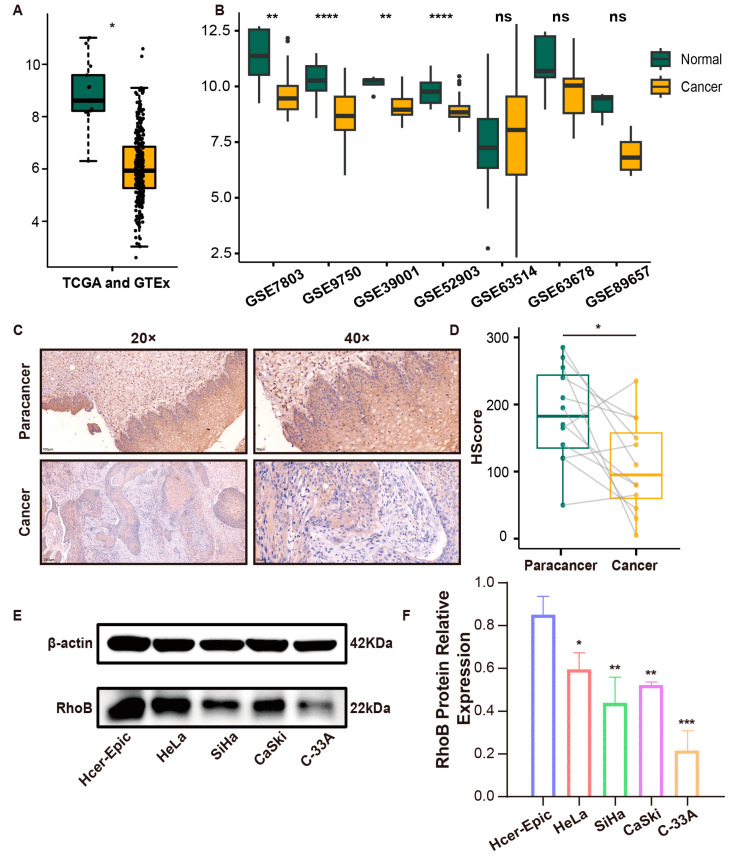
The expression of RhoB is downregulated in CC. (**A**) *RhoB* mRNA expression in normal cervix tissues and CC tissues of the TCGA and GTEx databases. (**B**) *RhoB* mRNA levels in GSE7803, GSE9750, GSE39001, GSE52903, GSE63514, GSE63678, and GSE89657 datasets. (**C**) Representative IHC images of RhoB expression in normal and CC tissues. (**D**) The HScore of RhoB between normal cervix tissues and CC tissues. (**E**,**F**) RhoB protein expression in normal cervix epithelial cells and CC cells. * *p* < 0.05, ** *p* < 0.01, *** *p* < 0.001, and **** *p* < 0.0001. ns indicates no significance.

As depicted in Figure 1A, *RhoB* mRNA levels exhibited a notable decrease in CC tissues relative to normal cervix tissues. Furthermore, the *RhoB* mRNA level in CC tissues was lower than normal cervical tissues across multiple GEO datasets, including GSE7803, GSE9750, GSE39001, and GSE52903 (Figure 1B). This expression of RhoB was further validated through an IHC experiment conducted on 12 paired CC and paracancer tissues (Figure 1C,D). The subsequent WB analyses also confirmed the downregulation of RhoB expression in cancer cells compared to that of Hcer-Epic cells (Figure 1E,F).

### 3.2. High RhoB Expression Is an Independent Risk Factor for Predicting CC Prognosis

**Figure 2:**
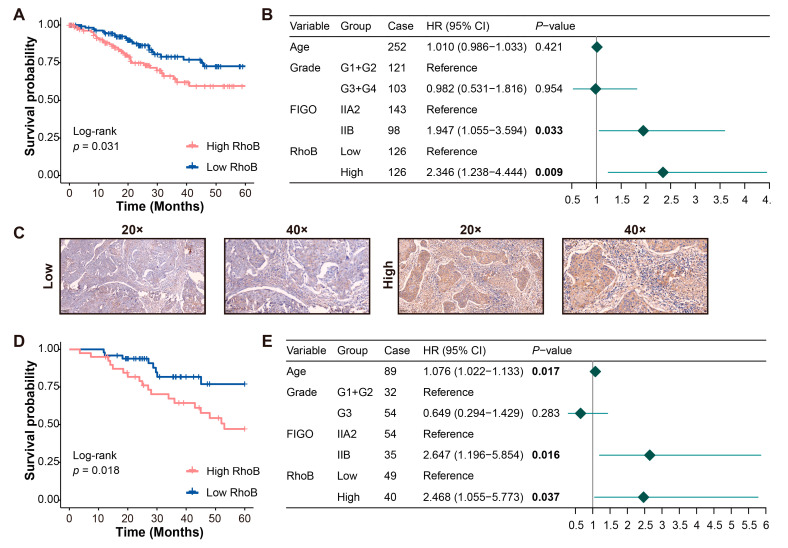
High RhoB expression is an independent risk factor for predicting CC prognosis. (**A**) Survival curves of overall survival based on the TCGA database. (**B**) A forest plot presents the results from the multivariate Cox proportional hazard regression model using TCGA data. (**C**) Representative IHC images show varying RhoB expression levels in 89 CC patients from Tongji Hospital. (**D**) Survival curves of OS of 89 CC patients. (**E**) A forest plot shows the multivariate Cox proportional hazards regression findings for 89 CC patients.

Based on the mRNA expression data of *RhoB* in squamous CC cells (*n* = 252) from TCGA, we analyzed the association between the *RhoB* mRNA levels and OS. A high *RhoB* expression was correlated with a shorter OS (*p* = 0.031) (Figure 2A). The multivariate Cox regression analysis identified RhoB as an independent prognostic factor for OS (*p* = 0.009) (Figure 2B). We further assessed the impact of RhoB protein expression on OS in 89 CC patients using the IHC experiment. Patients with an HScore ≥ 100 were categorized into the high-expression RhoB group (*n* = 40), while those with an HScore < 100 formed the low-expression RhoB group (*n* = 49) (Figure 2C). The survival analysis underscored a significant association between high RhoB expression and poor prognosis (*p* = 0.018) (Figure 2D). The multivariate Cox regression analysis further also identified high RhoB protein expression as an independent adverse prognostic factor for OS (*p* = 0.037) (Figure 2E).

### 3.3. RhoB Promotes the Malignant Phenotype of CC Cells In Vitro

**Figure 3:**
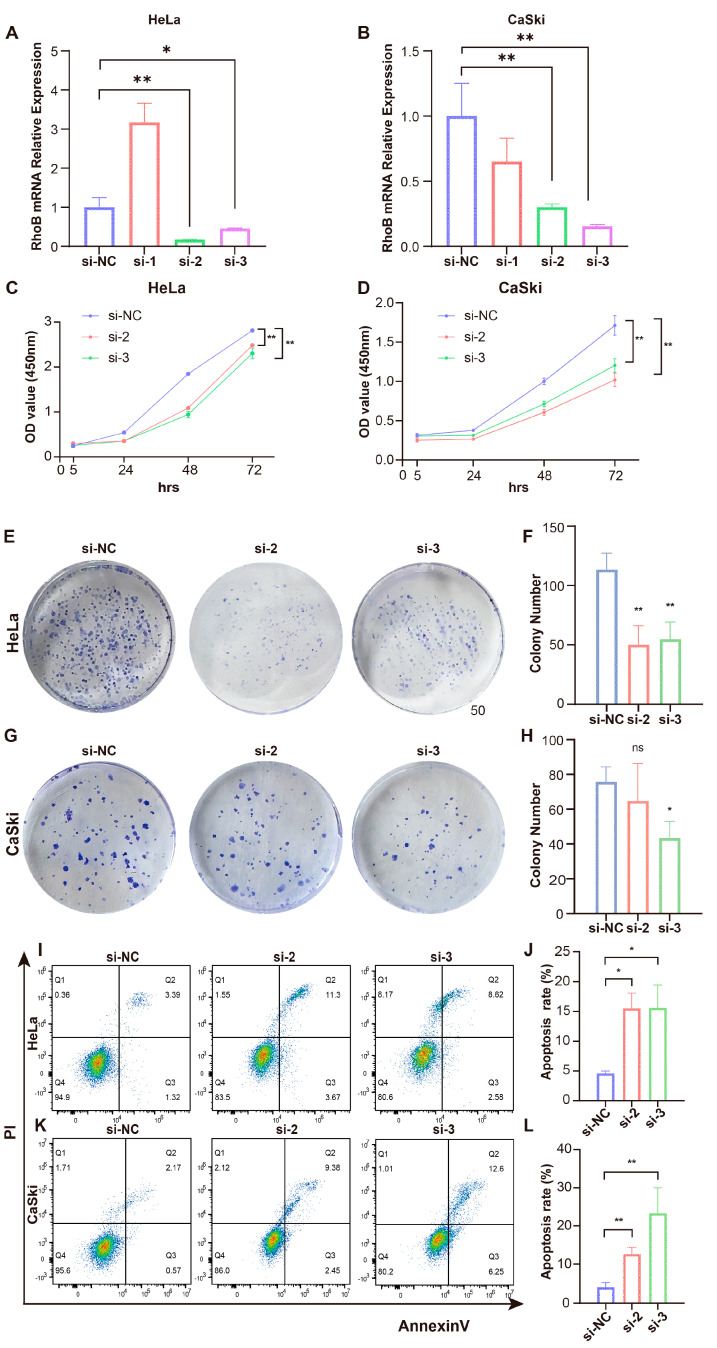
RhoB promotes the malignant phenotype of CC cells in vitro. (**A**,**B**) *RhoB* mRNA expression of HeLa and CaSki cells after transfecting three si-RhoB. (**C**,**D**) CCK-8 assays measured cell growth upon RhoB knockdown in HeLa and CaSki cells. (**E**,**F**) Colony formation assays determined the colony-forming capacity of CC cells following RhoB knockdown. (**G**,**H**) Apoptosis was evaluated by flow cytometry in HeLa and CaSki cells after RhoB knockdown. (**I**–**L**) Apoptosis was evaluated by flow cytometry after RhoB knockdown. * *p* < 0.05, ** *p* < 0.01, ns indicates no significance.

**Figure 4:**
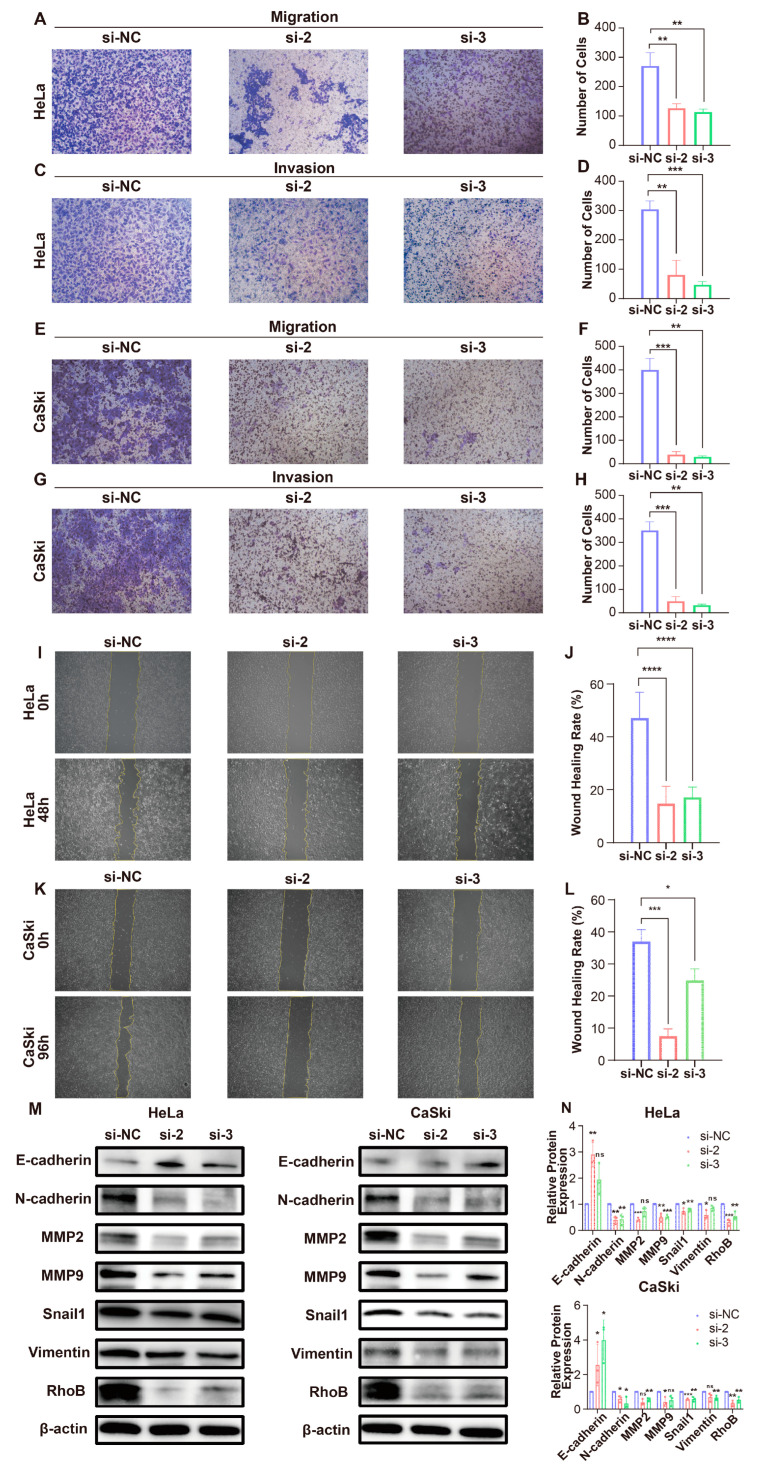
RhoB promotes the migration and invasion of CC cells. (**A**–**H**) The migration and invasion abilities of HeLa and CaSki cells were evaluated through transwell assays, following RhoB knockdown. (**I**–**L**) To examine the migration of CC cells after RhoB knockdown, wound healing tests were conducted. (**M**,**N**) WB analysis quantified the protein levels of EMT-related markers in HeLa and CaSki cells after RhoB knockdown * *p* < 0.05, ** *p* < 0.01, *** *p* < 0.001, and **** *p* < 0.0001, ns indicates no significance.

To investigate the function of RhoB in CC, we transfected three si-RhoBs into HeLa and CaSki cells to knock down RhoB expression. The effectiveness of this knockdown was confirmed through RT-qPCR and WB analyses (Figure 3A,B, Supplementary Figure S3A,B). Therefore, siRhoB-2 and siRhoB-3 were selected for subsequent experiments. The capacity for cell proliferation was initially evaluated using the CCK-8 assay. Following RhoB knockdown, the results show that the relative cell viabilities of HeLa and CaSki cells were much lower than those of the control group (Figure 3C,D). Additionally, the colony formation analysis revealed a decrease in colony number in HeLa and CaSki cell lines upon RhoB knockdown (Figure 3E–H). These findings suggest that RhoB can enhance the proliferation of CC cells. Flow cytometry was then used to examine how RhoB affects apoptosis in HeLa and CaSki cells, and the results show that apoptosis levels were higher after RhoB was knocked down (Figure 3I–L). Through transwell tests, we investigated how RhoB affects CC cell migration and invasion, and we found that the migration and invasion abilities of HeLa and CaSki cells were significantly reduced following RhoB suppression (Figure 4A–H). The outcomes of the wound healing assays were consistent with the transwell assays (Figure 4I–L).

### 3.4. RhoB Modulates Epithelial–Mesenchymal Transition (EMT) in CC Cell Lines

In order to elucidate the functional and pathway implications of RhoB enrichment in CC, we identified the top 500 genes exhibiting a high correlation with *RhoB* expression. The subsequent GO and KEGG analyses revealed close associations between RhoB and cadherin binding, extracellular matrix structural constituents, and the PI3K-Akt signaling pathway (Supplementary Figure S1). Through functional experiments, we found that knocking down RhoB significantly inhibits the invasion and migration of CC cells, and EMT plays a crucial role in the distant metastasis of CC. Therefore, we hypothesized that RhoB promotes EMT, which in turn increases these cells’ migration and invasion capabilities. To test this hypothesis, we examined the expression of EMT-related proteins after knocking down RhoB using WB. The WB results show that, in HeLa and CaSki cells, knocking down RhoB led to an increase in the expression of the epithelial marker E-cadherin, while the expression of N-cadherin, MMP2, MMP9, Vimentin, and Snail1 decreased (Figure 4M,N). These findings suggest that RhoB may promote the migration and invasion of CC cells through the EMT.

### 3.5. RhoB Promotes Cisplatin Sensitivity in CC

**Figure 5:**
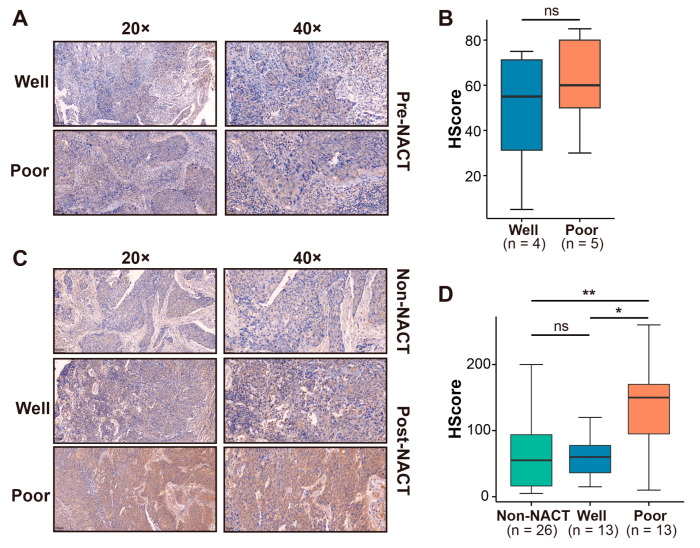
The expression of RhoB is upregulated in post-NACT CC tissues. (**A**,**B**) IHC images and HScore of RhoB expression of good responders and poor responders in pre-NACT CC tissues. (**C**,**D**) IHC images and HScore of RhoB expression of CC tissues of non-NACT and good responders and poor responders in post-NACT CC tissues. * *p* < 0.05, ** *p* < 0.01. ns indicates no significance.

**Figure 6:**
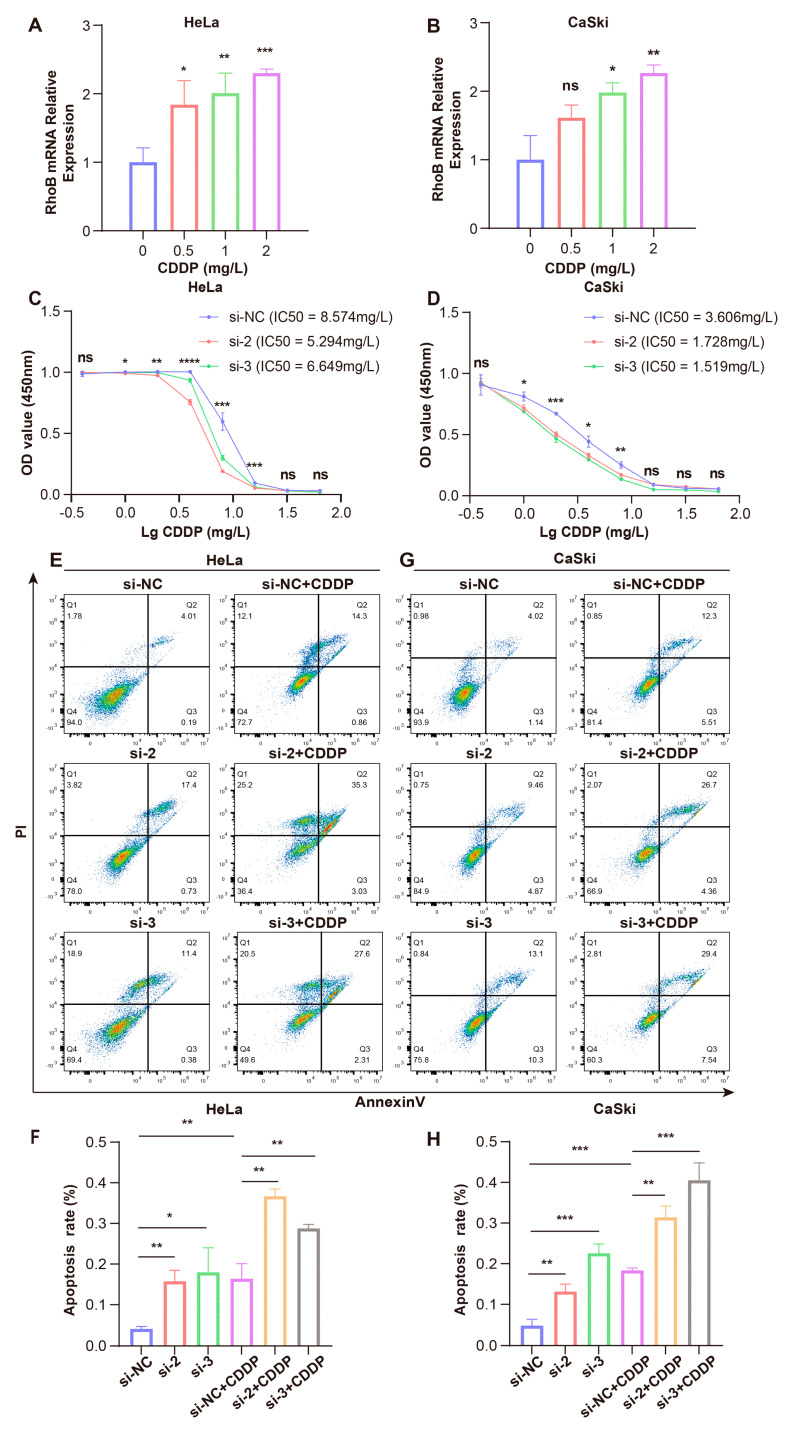
RhoB promotes cisplatin sensitivity in CC. (**A**,**B**) Dose-dependent increases in *RhoB* mRNA levels in HeLa and CaSki cells were measured via RT-qPCR. (**C**,**D**) The growth curves of HeLa and CaSki cells stimulated in differently dosed cisplatin for 48 h. (**E**–**H**) Apoptosis was evaluated by flow cytometry after 48 h of cisplatin treatment. * *p* < 0.05, ** *p* < 0.01, *** *p* < 0.001, and **** *p* < 0.0001. ns indicates no significant difference.

Our analysis revealed a significant upregulation of *RhoB* mRNA expression in post-NACT CC tissues compared to their pre-NACT tissues (Supplementary Figure S2). Additionally, RhoB protein expression was investigated in pre-NACT CC tissues (four good responders and five poor responders) using IHC experiments. The result exhibits that the IHC HScore in poor responders was higher than in good responders, but no statistically significance was identified (Figure 5A,B). We further explored the RhoB protein expression in CC tissues without NACT (*n* = 26) and post-NACT CC tissues (13 good responders and 13 poor responders) through IHC. We observed that RhoB protein levels in poor responders were notably elevated compared to CC tissues without NACT and good responders (Figure 5C,D). Next, we observed that the cisplatin treatment led to a dose-dependent increase in RhoB mRNA and protein levels in HeLa and CaSki cells, as evidenced by RT-qPCR and WB analysis (Figure 6A,B, Supplementary Figure S3C,D). Subsequently, HeLa cells transfected with siRhoB-2 and siRhoB-3 were cultured in the DMEM medium containing varying concentrations of cisplatin. CCK8 assays revealed that RhoB knockdown dose-dependently suppressed cell proliferation and decreased the IC50 values of cisplatin. Specifically, the IC50 values were recorded as 8.574 mg/L, 5.294 mg/L, and 6.649 mg/L in the control, siRhoB-2, and siRhoB-3 groups, respectively (Figure 6C). Similar trends were observed in CaSki cells, with IC50 values of 3.606 mg/L, 1.728 mg/L, and 1.519 mg/L in the control, siRhoB-2, and siRhoB-3 groups, respectively (Figure 6D). Furthermore, RhoB knockdown was found to augment cell apoptosis induced by cisplatin, as revealed by flow cytometry assays (Figure 6E–H).

## 4. Discussion

RhoB, a member of the Ras superfamily, is modulated by guanine nucleotide exchange factors and GTPase-activating proteins [26,27]. In mammals, the Rho family GTPase comprises 20 members classified into eight subgroups [28]. Rho proteins mainly include RhoA, RhoB, and RhoC. However, the three proteins play different biological roles. RhoA and RhoC promote tumor progression in various cancers. RhoB exhibits a dual role, functioning as both an oncogene and a tumor suppressor, depending on the stage of cancer development and progression [29,30]. However, the specific effects of RhoB on CC have not been fully determined. This research delves into the biological functions of RhoB and its relationship with cisplatin sensitivity in CC.

WB and IHC experiments confirmed that RhoB expression was low in CC cells and tissues. The result is consistent with many studies, including ovarian cancer, lung cancer, gastric cancer, and clear-cell renal cell cancer [31,32,33,34,35]. However, RhoB is upregulated in breast cancer [36]. Based on the data of TCGA and Tongji Hospital, we observed that CC patients with a high RhoB expression were associated with a shorter OS. Nevertheless, there was a poor prognosis in patients with low RhoB expression in lung cancer, gastric cancer, and pancreatic cancer patients [31,33,37].

In our study, RhoB was associated closely with actin binding, extracellular matrix structural constituents, and the PI3K-Akt signaling pathway through GO and KEGG analyses. Melina Livitsanou et al. found that the RhoB/Smad3 complex in the cytoplasm is involved in the EMT. In the presence of RhoB, Smad3 can downregulate the expression of E-cadherin and upregulate the fibronectin gene, indicating that the RhoB/Smad3 complex in the cytoplasm may play a role in the EMT [38]. In colorectal cancer, Kopsida et al. found RhoB was related to the DNA damage pathway, P53 pathway, AKT, and FOXM1 pathway [11,39]. The knockdown of RhoB in HeLa and CaSki cells showed decreased tumor cell proliferation, increased apoptosis, and decreased migration and invasion, suggesting that RhoB may exert a cancer-promoting influence in CC. In prostate cancer, the overexpression of RhoB increased cell activity and increased migration ability, suggesting that RhoB has a cancer-promoting effect, which is consistent with our results [40].

We found that RhoB expression was significantly elevated after chemotherapy. The IHC experiments showed that RhoB expression was significantly increased in the tissues of patients who responded poorly to chemotherapy compared with those who did not receive chemotherapy and those who responded well to chemotherapy. In HeLa and CaSki cells, RhoB expression increased with higher concentrations of cisplatin. The IC50 values of HeLa cells and CaSki cells were significantly decreased after RhoB knockdown, suggesting that the knockdown of RhoB expression increased the cells’ sensitivity to cisplatin. Furthermore, RhoB knockdown could enhance cell apoptosis triggered by cisplatin. In colorectal cancer, Kopsida et al. also found increased sensitivity to 5-FU and oxaliplatin after RhoB knockdown [39].

In conclusion, RhoB promotes the development and progression in CC. A downregulated RhoB expression increases the sensitivity to cisplatin in CC cells. These suggest that RhoB can be used as a therapeutic target for CC and a predictive target for clinical cisplatin resistance.

## Data Availability

Public databases used in this study can be found in The Cancer Genome Atlas (https://portal.gdc.cancer.gov) (accessed on 1 February 2024) and the Gene Expression Omnibus (https://www.ncbi.nlm.nih.gov/geo/) (accessed on 1 February 2024). The accession number of paired pre-NACT and post-NACT tissues RNA-seq data in this study is SRP173984.

## Supplementary Materials

The following supporting information can be downloaded at: https://www.mdpi.com/article/10.3390/genes15091186/s1. Supplementary Figure S1. GO and KEGG analyses. (A) GO analysis. (B) KEGG analysis; Supplementary Figure S2. (A) The volcano plot of differentially expressed genes for paired pre-NACT and post-NACT cervical cancer tissues. *RhoB* is highlighted in the plot. (B) The box plot of *RhoB* mRNA expression for 14 pairs pre-NACT and post-NACT cervical cancer tissues; Supplementary Figure S3. (A,B) RhoB protein expression of HeLa and CaSki cells after transfecting three si-RhoB. (C,D) The protein level of RhoB in HeLa and CaSki cells with increased doses of cisplatin through Western blot; Table S1: The number of normal cervix tissues and cervical cancer tissues of TCGA and GTEx databases and 7 GEO datasets; Table S2: Clinical characteristics of cervical cancer patients from TCGA database and Tongji Hospital; Table S3: Sequences for RT-qPCR and gene silencing.
