# Supplementary material for: Downregulation of RhoB Inhibits Cervical Cancer Progression and Enhances Cisplatin Sensitivity
Source: Genes (Basel). 2024 Sep 10;15(9):1186. doi: 10.3390/genes15091186 (PMC11431011; doi:10.3390/genes15091186)
Supplement: Supplementary file 1 [file genes-15-01186-s001.zip › Supplementary Figure S2.pdf]

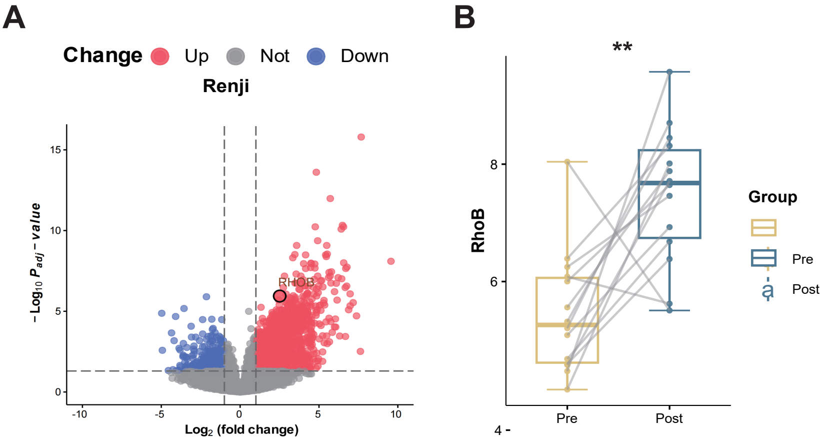

Supplementary Figure S2. (A) The volcano plot of differentially expressed genes for paired pre-NACT and post-NACT cervical cancer tissues. RhoB was highlight in the plot. (B) The box plot of RhoB mRNA expression for 14 pairs pre-NACT and post-NACT cervical cancer tissues.
