# Supplementary material for: Downregulation of RhoB Inhibits Cervical Cancer Progression and Enhances Cisplatin Sensitivity
Source: Genes (Basel). 2024 Sep 10;15(9):1186. doi: 10.3390/genes15091186 (PMC11431011; doi:10.3390/genes15091186)
Supplement: Supplementary file 1 [file genes-15-01186-s001.zip › Supplementary Figure S3.pdf]

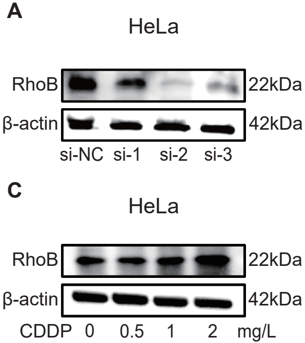


Supplementary Figure S3. (A-B) RhoB protein expression of HeLa and CaSki cells after transfecting three si-RhoB. (C-D)The protein level of RhoB in HeLa and CaSki cells with increased doses of cisplatin through western blot.
