# Supplementary figures and images for: Downregulation of RhoB Inhibits Cervical Cancer Progression and Enhances Cisplatin Sensitivity
Source: Genes (Basel). 2024 Sep 10;15(9):1186. doi: 10.3390/genes15091186 (PMC11431011; doi:10.3390/genes15091186)

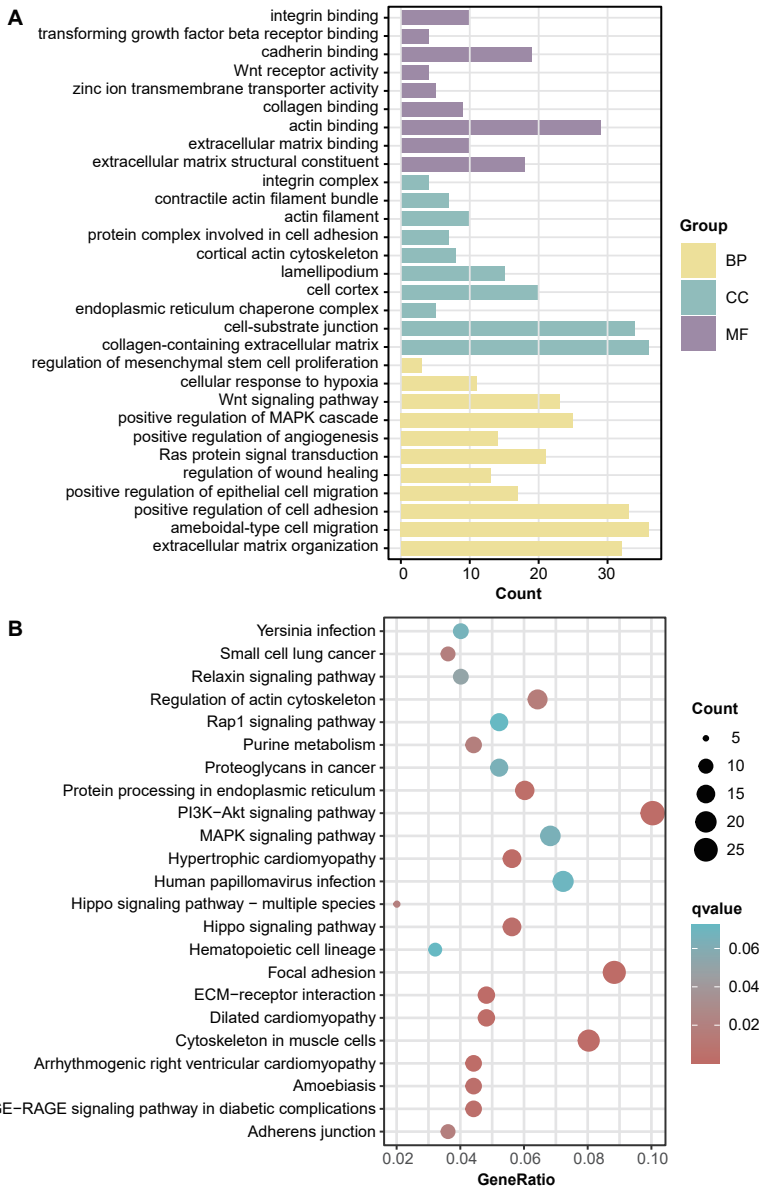

Supplementary Figure S1. GO and KEGG analysis. (A) GO analysis. (B) KEGG analysis.

Supplement: Supplementary file 1 [file genes-15-01186-s001.zip › Supplementary Figure S1.pdf]
